# Delta‐Cell Area is Unchanged in Human Pregnancy: Evidence From Immunohistochemistry

**DOI:** 10.1111/boc.70067

**Published:** 2026-05-06

**Authors:** Faheem Seedat, Neva Kandzija, Edward Drydale, Katie Holden, James Bancroft, John A. Todd, M. Irina Stefana, Manu Vatish

**Affiliations:** ^1^ Centre for Human Genetics, Nuffield Department of Medicine University of Oxford Oxford UK; ^2^ Nuffield Department of Women's and Reproductive Health University of Oxford Oxford UK

**Keywords:** delta cells, gestational diabetes, human islets, pancreatic islet, pregnancy, somatostatin

## Abstract

**Aim:**

Pancreatic islet *δ*‐cells produce somatostatin, a paracrine regulator of insulin and glucagon secretion within islets. Although adaptive changes in *α*‐ and *β*‐cell populations during pregnancy have been described in both animals and humans, data on *δ*‐cell plasticity are sparse and entirely lacking in human pregnancy. We aimed to determine whether pancreatic islet *δ*‐cell mass undergoes morphological adaptation during human pregnancy.

**Methods and results:**

Formalin‐fixed paraffin‐embedded pancreatic tissue from pregnant (*n* = 7) and non‐pregnant (*n* = 7) donors was analysed. Sections were immunolabelled for somatostatin to identify *δ* cells, and whole‐slide quantitative analysis was performed using an unbiased automated imaging pipeline. *δ*‐cell area was measured across the entire pancreatic sections and compared between groups. In contrast to previously reported expansion of *α*‐ and *β*‐cell populations in pregnancy, *δ*‐cell area was not significantly different between pregnant and non‐pregnant donors. No quantitative architectural alterations in *δ*‐cell distribution within islets were observed.

**Conclusion:**

Pancreatic *δ*‐cell area does not increase during human pregnancy. These findings demonstrate that endocrine cell plasticity within the maternal pancreas is selective and does not uniformly involve all islet cell subtypes.

## Graphical abstract

Pancreatic δ‐cell area is unchanged in human pregnancy despite well‐described expansion of *α*‐ and *β*‐cell populations. Using whole‐slide quantitative immunohistochemistry, we show that *δ*‐cell area and islet architecture remain stable, highlighting the selective nature of endocrine cell plasticity in the maternal pancreas.

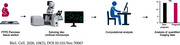

**Abbreviations**
FFPE: Formalin‐fixed paraffin‐embedded
GLP‐1: Glucagon‐like peptide‐1
HIPAA: Health Insurance Portability and Accountability Act
IHC‐IF: FFPE pancreatic tissue sections labelled by immunofluorescence
nPOD: Network for Pancreatic Organ Donors with Diabetes

## 1 Introduction

During pregnancy, many organ systems adapt to support foetal growth and prepare the mother for parturition (Sferruzzi‐Perri et al. 2020). The pancreatic islets of Langerhans also undergo functional and structural changes to meet the increased metabolic demands of gestation (Salazar‐Petres and Sferruzzi‐Perri 2022). Notably, *α*‐ and *β*‐cell mass increases in pregnancy while insulin production rises early in pregnancy and approximately doubles by term (Powe et al. 2019, Butler et al. 2010, Qiao et al. 2022, Seedat et al. 2025).

δ cells, an often‐overlooked population of islet cells, secrete somatostatin and play a role in intra‐islet paracrine signalling by modulating hormone secretion from neighbouring *α*‐ and *β*‐cells (Huising 2020, Hill and Hill 2024). In rodents, pregnancy induces marked islet remodelling, particularly in *α*‐ and *β*‐cell populations (Salazar‐Petres and Sferruzzi‐Perri 2022, Qiao et al. 2022, Hill and Hill 2024). In pregnant mice, the transcriptome of *δ*‐cells has been studied by single‐cell RNA sequencing and changes in immune and signalling pathways reported (Chung et al. 2023); however, enrichment of classical endocrine‐related pathways was not observed (Chung et al. 2023). The only direct morphological data related to *δ* cells in pregnancy come from a single study in pregnant rats with diabetes, which reported no significant change in *δ*‐cell area relative to total islet mass despite hyperglycaemia (Gallego et al. 2018). Overall, data on *δ*‐cell adaptation in pregnancy are limited in rodents, and no published data are currently available in humans. This is largely due to the difficulty of accessing well‐preserved human pancreatic tissue, which can only be acquired post‐mortem, representing a major limitation in islet research, particularly with respect to pregnant donors from which samples are exceptionally rare. Post‐mortem pancreatic tissues are degraded by autodigestion, but the Network for Pancreatic Organ Donors with Diabetes (nPOD) biorepository provides high‐quality, rapidly preserved pancreatic samples from pregnant donors, enabling reliable structural analyses of islets (Campbell‐Thompson et al. 2012).

We recently characterised human islet adaptations during pregnancy using rare pancreatic tissue from pregnant organ donors obtained from the nPOD biorepository. We observed significant expansion of *α*‐ and *β*‐cell populations, altered expression of prolactin and serotonin 2B receptors, changes in islet architecture, and increased glucagon‐like peptide‐1 (GLP‐1) expression in α cells (Seedat et al. 2025).

In the present study, we extend this work by quantifying *δ*‐cell abundance in the same donor cohort of pregnant women and non‐pregnant controls. Pancreatic sections were labelled by immunohistochemistry and analysed using a previously described automated image analysis pipeline (Seedat et al. 2025). By comparing pregnant and non‐pregnant groups, we aimed to determine whether *δ*‐cell populations undergo structural remodelling in human pregnancy. Providing insight into the plasticity and potential role of *δ* cells in this context is essential for a complete understanding of islet adaptation in pregnancy.

## 2 Methods

### 2.1 Donor Tissues

Formalin‐fixed paraffin‐embedded (FFPE) human pancreatic tissues (4 µm thickness) were obtained from the nPOD biorepository at the University of Florida. FFPE pancreatic tissue from seven third‐trimester pregnant and seven non‐pregnant organ donors along with their corresponding donor data, was provided. Donor characteristics are described in detail in our previous publication (Seedat et al. 2025). Donors’ identities were anonymised in accordance with Health Insurance Portability and Accountability Act (HIPAA) regulations (USA). Ethical approval was granted by the South Central—Oxford A Research Ethics Committee (REFS 18/SC/0559).

### 2.2 Immunofluorescence Labelling of *δ* Islet Cells and *δ*‐Cell Area Quantification

FFPE pancreatic tissue sections were labelled by immunofluorescence (IHC‐IF) as previously described (Seedat et al. 2025) for somatostatin (Santa Cruz Biotechnology, cat.no. sc‐55565), insulin (Agilent, cat.mo. IR00261‐2), and glucagon (Abcam, cat.no. ab92517) to allow identification of *δ*, *β* and *α* cells, respectively. Nuclei were labelled using DAPI (1 µg/mL; Thermo Fischer Scientific, cat. no. 62248).

Whole‐section imaging and quantitative image analysis were performed using the same previously validated image acquisition and analysis pipeline (Seedat et al. 2025). All image quantification was conducted blinded to group status. *δ*‐cell area was defined as the somatostatin‐positive region within each islet. δ‐cell measurements were normalised in three ways: to total tissue section area (fractional area), to the number of islets per tissue section (mean area) and to total islet area per tissue section (proportion of islet area). Pregnant donors were compared to non‐pregnant controls, best matched by age and race.

### 2.3 Statistical Analysis

Statistical comparisons between pregnant and non‐pregnant donors were performed using unpaired two‐tailed t‐tests for normally distributed data, or two‐sided Mann–Whitney tests for non‐parametric data. Results are presented as mean ± SEM unless otherwise stated. Statistical significance was defined as *P* < 0.05. Outliers were assessed using the ROUT method (Robust Regression and Outlier Removal) with a *Q* value of 1%. Identified outliers were reviewed for potential biological or technical explanations prior to analysis. Primary analyses included all data points, and sensitivity analyses were conducted with outliers excluded to assess the robustness of the findings.

## 3 Results

### 3.1 *δ*‐Cell Area is Unchanged in Human Pregnancy

**Figure 1:**
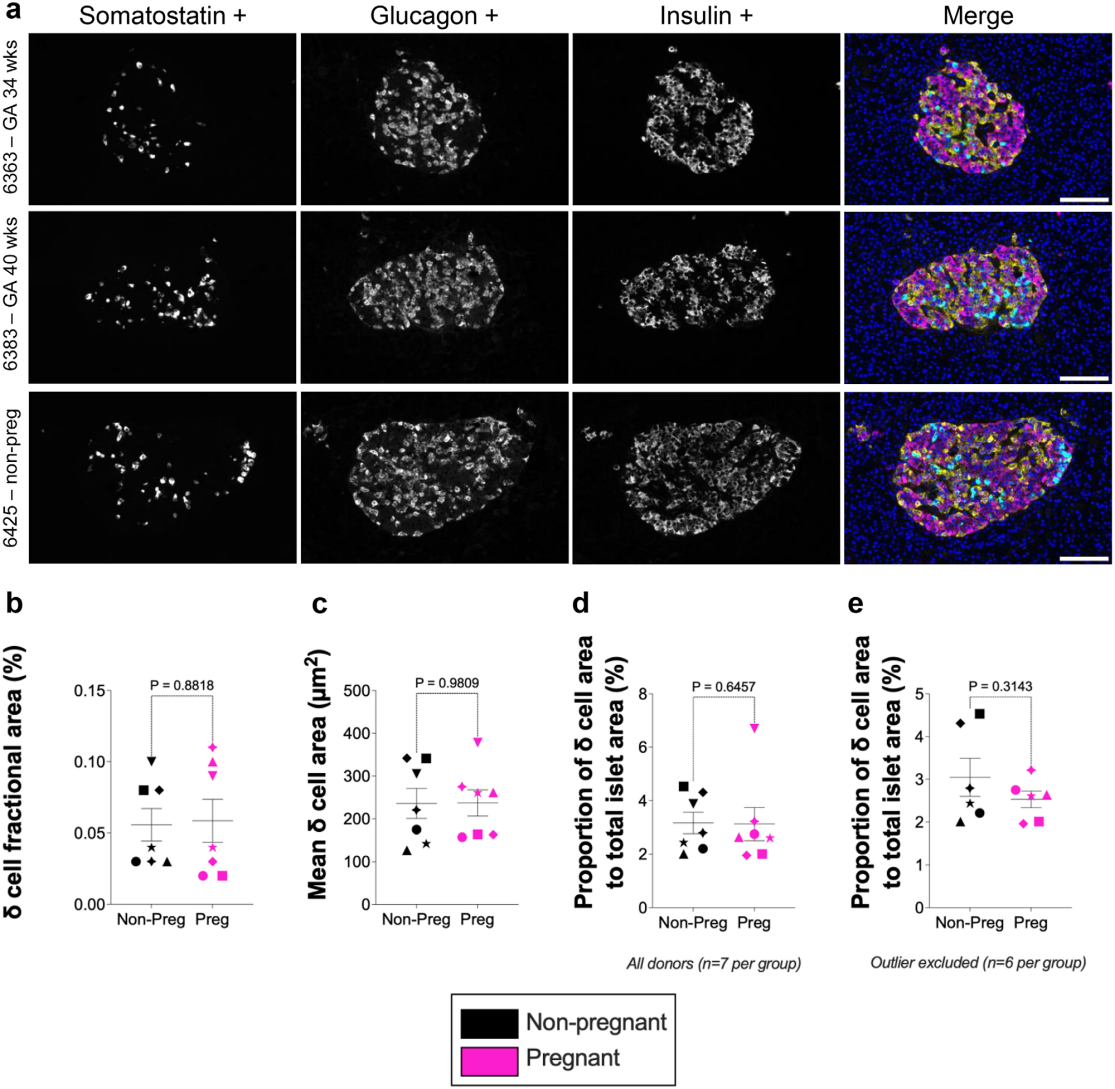
Immunofluorescence labelling of human pancreas tissue (IHC‐IF) and quantification of *δ*‐cell metrics, comparing pregnant and non‐pregnant groups. (a) IHC‐IF analysis of human pancreatic sections from pregnant women at different gestational ages and a non‐pregnant control sample. *α*‐, *β*‐ and *δ*‐cells are shown. The grayscale images represent the individual channels for somatostatin, glucagon and insulin. Somatostatin (cyan), glucagon (yellow), insulin (magenta) and nuclei (DAPI, blue) are shown in the merged channel. Scale bar = 200 µm. (b) Fractional δ cell area (measured area as a percentage of total tissue area), (c) mean *δ* cell area (measured area divided by number of islets per tissue section) (d) proportions of δ cells relative to whole islets (measured area as a percentage of total whole islet area) (e) sensitivity analysis of proportions of δ cells relative to whole islets after excluding one statistical outlier (ROUT, Q = 1%) and its matched control are shown. Symbols in each figure correspond to individual donors as previously described in Seedat et al. (2025). The pregnant group (*n*  =  7 biological replicates) was compared to the non‐pregnant control group (*n* = 7 biological replicates). Each biological replicate represents an independent human donor. Data are presented as mean ± SEM. Normally distributed data were analysed using a two‐sided unpaired Student's *t*‐test; non‐parametric data were analysed using a two‐sided Mann–Whitney test. Exact *P* values for each comparison are shown in the figure. Statistical significance was defined as *P* < 0.05.

Islets, defined by *α*‐, *β*‐ and *δ*‐cell marker expression, were readily identified across the pancreatic tissue (Figure 1a). The automated image analysis pipeline was applied to the whole‐pancreas sections to enable unbiased quantification of labelled islet cell populations. In contrast to the previously reported expansion of *α*‐ and *β*‐cell areas during pregnancy (Seedat et al. 2025), *δ*‐cell area showed no significant change between pregnant and non‐pregnant groups.

Specifically, *δ*‐cell fractional area (i.e., *δ*‐cell area as a percentage of total tissue section area) did not differ between groups (0.059 ± 0.02% vs. 0.053 ± 0.01%, *p*  =  0.8818; Figure 1b). Similarly, the mean *δ*‐cell area per islet (i.e., total *δ*‐cell area divided by the number of islets) was unchanged (237.1 ± 30.76 µm^2^ vs. 244.7 ± 38.84 µm^2^, *p*  =  0.9809; Figure 1c). The proportion of islet area occupied by *δ* cells (i.e., *δ*‐cell area as a percentage of total islet area) also showed no difference between groups (3.126 ± 0.62% vs. 3.43 ± 0.54%, *p*  =  0.6457; Figure 1d).

### 3.2 Sensitivity Analysis

To assess the robustness of the result for the proportion of islet area occupied by *δ* cells comparison, we performed a sensitivity analysis excluding one statistical outlier (identified using the ROUT method, *Q * =  1%) and its matched control. This secondary analysis also showed no significant difference in *δ*‐cell proportion of islet area (pregnant: 2.53 ± 0.19% vs. non‐pregnant: 3.05 ± 0.45%, *p*  =  0.3143; Figure 1e), indicating that findings were not driven by a single donor and remained consistent after outlier exclusion.

## 4 Discussion

In this study, we investigated whether *δ*‐cell abundance is altered in human pregnancy by extending our previous analysis of rare pancreatic tissue from pregnant donors (Seedat et al. 2025). While our earlier work identified significant expansion of *α*‐ and *β*‐cell area during pregnancy (Seedat et al. 2025), here we show that *δ*‐cell area does not differ between pregnant and non‐pregnant donors, whether assessed as fractional area, mean area per islet, or a proportion of total islet area. This finding in the proportion of total islet area comparison remained consistent in a secondary sensitivity analysis after excluding a statistical outlier, reinforcing the robustness of the result.

The unchanged abundance of *δ* cells in human pregnancy suggests limited structural adaptation of this cell population. This observation is also noted in animal studies: in pregnant mice, single‐cell RNA sequencing revealed only modest transcriptional changes in *δ* cells without enrichment of classical endocrine remodelling pathways (Chung et al. 2023). Additionally, the only available morphological study of δ cells in rats—conducted in the context of diabetic pregnancy—reported no significant change in *δ*‐cell area relative to total islet mass (Gallego et al. 2018). Taken together, these findings suggest that *δ* cells may be relatively resistant to both transcriptional and structural adaptation during pregnancy, in contrast to the well‐described expansion of *α*‐ and *β*‐cell populations.

The functional role of *δ* cells in pregnancy, however, remains unclear. Somatostatin has paracrine effects within islets, modulating insulin and glucagon secretion from neighbouring *α* and *β* cells, respectively (Huising 2020, Hill and Hill 2024). Even without morphological expansion, *δ* cells may adapt during pregnancy to contribute to the dynamic regulation of glucose homeostasis through mechanisms other than changes in cell size or number. These may include local alterations in cellular connectivity and paracrine signalling, or in somatostatin secretory function. Given their relatively low abundance, such functional or ultrastructural adaptations may allow *δ* cells to maintain effective regulation of islet hormone secretion without requiring substantial expansion in cell mass. These processes cannot be assessed using immunohistochemistry but may contribute to preservation of islet function, despite the absence of detectable changes in *δ*‐cell area in the present study, and require further investigation.

This study has several limitations. The rarity of high‐quality pancreatic tissue from pregnant organ donors constrained our sample size and introduced unavoidable biological heterogeneity. Although donors were matched as closely as possible, variation in ethnicity, BMI, gestational age and other clinical parameters may have introduced confounding effects. Given that *δ* cells comprise only ∼3%–5% of total islet area, modest absolute increases in *δ*‐cell mass occurring alongside the more substantial expansion of *α*‐ and *β*‐cell populations may be difficult to detect. As such, the stability of *δ*‐cell proportional area does not exclude subtle increases in absolute *δ*‐cell area that fall below the resolution of the current analysis. A limitation of this study is the use of section‐based immunohistochemistry, which does not permit high‐resolution or single‐cell quantification of *δ*‐cell morphology, and therefore may underestimate subtle changes in *δ*‐cell number or size. Larger cohorts and higher‐resolution approaches may be required to resolve subtle *δ*‐cell changes. A further limitation of this study is the use of rare archived human FFPE pancreatic tissue, which precludes high‐resolution spatial and functional analyses, including direct assessment of cell proliferation and apoptosis, three‐dimensional islet architecture and *δ*‐cell positioning relative to other endocrine cell types, as well as approaches such as stimulus–secretion assays or single‐cell and spatial transcriptomics. Donors with gestational diabetes mellitus (GDM) were included in the cohort; however, given the small sample size, particularly in the context of the low abundance of *δ* cells, subgroup analysis was not performed, as any conclusions would not be sufficiently robust.

Finally, our analysis focused on structural measures, and we cannot exclude the possibility of functional *δ*‐cell adaptation that would not be apparent from morphology alone.

In conclusion, our data suggest that *δ*‐cell area does not change significantly during human pregnancy, in contrast to the known expansion of *α* and *β* cells. These findings add to a limited body of literature and provide new insight into the nuances of islet remodelling in gestation. Further studies integrating transcriptomic, proteomic, and functional analyses will be necessary to fully elucidate the role of δ cells in human pregnancy.

## Author Contribution

F.S. and M.I.S. conceptualised the study, designed experiments, and directed the research. F.S. performed the major experiments and drafted the initial manuscript. J.B., K.H. and E.D. assisted with image acquisition and image analysis. J.T., N.K. and M.V. contributed intellectually to the overall study design and manuscript editing. All authors read and reviewed the final manuscript.

## Funding

This research was supported by the Wellcome Trust (grants 091157/Z/10/Z and 107212/Z/15/Z), the Juvenile Diabetes Research Foundation (JDRF; grants 9 2011 253, 5 SRA 2015 130 A N, and 4 SRA 2017 473 A N), and the 2021 EASD–Novo Nordisk Foundation Diabetes Prize for Excellence, awarded to John A Todd and the Diabetes and Inflammation Laboratory, University of Oxford.

## Conflicts of Interest

The authors declare no conflicts of interest.

## Data Availability

The datasets analysed in this paper are available from the authors upon reasonable request.
